# Integrated Genotypic Analysis of Hedgehog-Related Genes Identifies Subgroups of Keratocystic Odontogenic Tumor with Distinct Clinicopathological Features

**DOI:** 10.1371/journal.pone.0070995

**Published:** 2013-08-07

**Authors:** Yasuyuki Shimada, Ken-ichi Katsube, Yuji Kabasawa, Kei-ichi Morita, Ken Omura, Akira Yamaguchi, Kei Sakamoto

**Affiliations:** 1 Section of Oral and Maxillofacial Surgery, Department of Oral Restitution, Division of Oral Health Sciences, Graduate School of Medical and Dental Sciences, Tokyo Medical and Dental University, Tokyo, Japan; 2 Oral Pathology, Department of Oral Restitution, Division of Oral Health Sciences, Graduate School of Medical and Dental Sciences, Tokyo Medical and Dental University, Tokyo, Japan; 3 Global Center of Excellence Program, International Research Center for Molecular Science in Tooth and Bone Diseases, Tokyo Medical and Dental University, Tokyo, Japan; 4 Department of Advanced Molecular Diagnosis and Maxillofacial Surgery, Hard Tissue Genome Research Center, Tokyo Medical and Dental University, Tokyo, Japan; Philipps University, Germany

## Abstract

Keratocystic odontogenic tumor (KCOT) arises as part of Gorlin syndrome (GS) or as a sporadic lesion. Gene mutations and loss of heterozygosity (LOH) of the hedgehog receptor PTCH1 plays an essential role in the pathogenesis of KCOT. However, some KCOT cases lack evidence for gene alteration of *PTCH1*, suggesting that other genes in the hedgehog pathway may be affected. PTCH2 and SUFU participate in the occurrence of GS-associated tumors, but their roles in KCOT development are unknown. To elucidate the roles of these genes, we enrolled 36 KCOT patients in a study to sequence their entire coding regions of *PTCH1*, *PTCH2* and *SUFU*. LOH and immunohistochemical expression of these genes, as well as the downstream targets of hedgehog signaling, were examined using surgically-excised KCOT tissues. *PTCH1* mutations, including four novel ones, were found in 9 hereditary KCOT patients, but not in sporadic KCOT patients. A pathogenic mutation of *PTCH2* or *SUFU* was not found in any patients. LOH at *PTCH1* and *SUFU* loci correlated with the presence of epithelial budding. KCOT harboring a germline mutation (Type 1) showed nuclear localization of GLI2 and frequent histological findings such as budding and epithelial islands, as well as the highest recurrence rate. KCOT with LOH but without a germline mutation (Type 2) less frequently showed these histological features, and the recurrence rate was lower. KCOT with neither germline mutation nor LOH (Type 3) consisted of two subgroups, Type 3A and 3B, which were characterized by nuclear and cytoplasmic GLI2 localization, respectively. Type 3B rarely exhibited budding and recurrence, behaving as the most amicable entity. The expression patterns of CCND1 and BCL2 tended to correlate with these subgroups. Our data indicates a significant role of *PTCH1* and *SUFU* in the pathogenesis of KCOT, and the genotype-oriented subgroups constitute entities with different potential aggressiveness.

## Introduction

Gorlin syndrome (GS) is a rare autosomal-dominant genetic disease, characterized by developmental disorders, such as falx cerebri calcification, palmoplantar pits and rib deformities. Patients are also predisposed to several neoplasms, including basal cell carcinoma (BCC), medulloblastoma and fibroma of ovary or heart [1], [2]. Mutations in *PTCH1*, the homologue of drosophila segment polarity gene *patched*, have been found in GS patients, and this spectrum of neoplasms is thought to arise due to a malfunction of PTCH1 [3], [4]. The Patched protein is a 12-pass transmembrane receptor of Hedgehog, and it behaves as a tumor suppressor [5]. Binding with Hedgehog relieves the inhibitory effect of Patched on the latent activity of Smoothened. This results in activation of the transcription factor *cubitus interruptus* (Ci), whose mammalian homologue is Gli, which mediates the hedgehog target gene expression [6]. Gli regulates a broad range of downstream gene targets such as *PTCH1*, *Cyclin D (CCND)*, *BCL2* and *FOXM1* [7], [8], [9].

Jaw cysts are another major manifestation that frequently arises in GS patients [10]–[13]. Theses cysts exhibit specific microscopic features such as parakeratinized squamous epithelial lining and a palisaded basal layer [14]. The lesion with these histological features develops also sporadically in non-GS patients and shows potential for a locally destructive behavior and a higher recurrence rate than the other jaw cysts. Accordingly, the WHO working group recognized this cystic lesion as a tumor and recommended the use of the term keratocystic odontogenic tumor (KCOT) [14].

In Knudson’s two-hit theory of tumorigenesis, which was developed to understand the pathogenesis of retinoblastoma, the tumor is thought to arise due to loss of function at both loci of the tumor suppressor gene, *RB1* [15]. In a familial retinoblastoma patient, the mutation of *RB1* (the first hit) has been germinally transmitted, and any postnatal insult on the unaffected allele (the second hit) would trigger tumor formation, explaining the high incidence and the early onset of retinoblastoma and predisposition to other tumors such as osteosarcoma [16]. Loss of heterozygosity (LOH) at the tumor suppressor gene locus is a common hallmark of tumors [17] and is suggested as a key mechanism for malfunction of the gene as well as a point mutation in tumor development [18]. The pathogenesis of GS-associated tumors can also be understood according to the two-hit concept of *PTCH1* [18]–[20]. More than 90% of GS-associated BCC [20], [21] and 50–90% of sporadic BCC [22]–[24] have LOH at the *PTCH1* locus, suggesting that allelic loss is a key event that underlies the development of both GS-associated and sporadic BCC. Similarly, 50–80% of GS-associated KCOT carries LOH at the *PTCH1* locus, and LOH is also detected in about 30% of sporadic KCOT [18], [19]. Although these results have demonstrated the essential role of *PTCH1* in the occurrence of BCC and KCOT, there are cases in which no alteration of *PTCH1* is detected. 10–20% GS patients have no *PTCH1* mutations, and *PTCH1* LOH is not always detected in KCOT. This suggests that other genes in the hedgehog signaling pathway may be affected in these diseases.

*PTCH2* is another homologue of drosophila *patched*, which shares a 56% overall amino acid sequence identity to *PTCH1*. A germline mutation of *PTCH2* was identified in a familial GS pedigree that had no mutation in *PTCH1* [25]. Somatic mutations of *PTCH2* were found in sporadic BCC and medulloblastoma [26]. These results suggest that *PTCH2* can act in a similar way as *PTCH1* in the development of these tumors.

*Suppressor of fused* is a negative regulator of the hedgehog pathway. Its mammalian homologue, Sufu, binds to Gli and antagonizes its activity by sequestering Gli proteins in the cytoplasm or inhibiting Gli transcriptional activity in the nucleus [27], [28]. Missense or nonsense mutations of *SUFU* have been noted in GS patients [29], [30], and a high incidence of KCOT-like jaw lesions and BCC-like skin tumors have been observed in *Sufu* ^+/−^ mice [31]. These results suggest that malfunction of PTCH2 and SUFU may cause the symptoms of GS, but their contribution to KCOT development in humans remains unclear.

We have hypothesized the following: 1) *PTCH2* and *SUFU* may be affected in GS patients who have no *PTCH1* mutation; 2) sporadic KCOT patients may be predisposed to KCOT because of heterozygous germline mutations of *PTCH1*, *PTCH2* or *SUFU*, whose phenotypes are too weak to present with any other symptoms; and 3) sporadic KCOT that has no alteration in *PTCH1* locus may develop as a result of alterations in *PTCH2* or *SUFU* locus.

**Figure 1:**
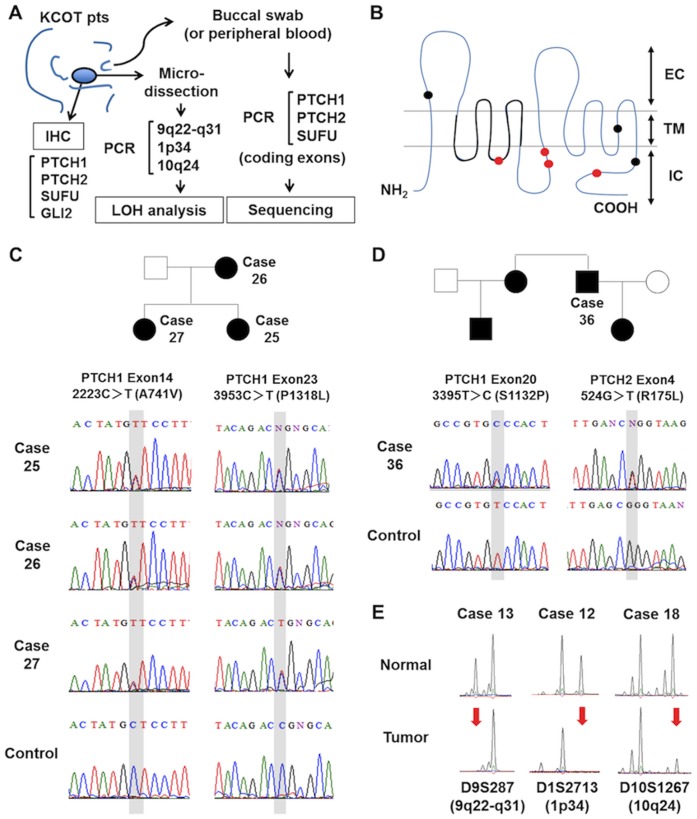
Analyses of gene mutation and LOH. A) Experimental scheme. Mutational analysis was conducted on patients with KCOT. LOH analysis and immunohistochemical analysis were performed using surgically excised KCOT specimens. B) Predicted secondary structure of PTCH1, showing the location of mutations. The black circles designate previously-reported mutations, and the red circles designate novel mutations. EC, Extracellular; IC, Intracellular; TM, Transmembrane domain. The thick black line represents a sterol-sensing domain. C) A family tree of Case 25, 26, 27 and the electropherograms showing the *PTCH1* mutations. Two missense mutations (2223C>T, 3953C>T) were found in the *PTCH1* coding region in this pedigree. D) A family tree of Case 36 and the electropherogram showing missense mutations of *PTCH1* (3395T>C) and *PTCH2* (524G>T). E) Electropherograms showing LOH of *PTCH1* (D9S287, Case 12), *PTCH2* (D1S2713, Case 13) and *SUFU* (D10S1267, Case 18) (arrows). Normal, normal cells; Tumor, KCOT cells.

The present study was initiated in order to clarify the contribution of *PTCH2* and *SUFU* to KCOT development. Mutational analysis of *PTCH1*, *PTCH2* and *SUFU* was conducted on patients with KCOT, and LOH analysis and immunohistochemical analysis were performed on the surgically-excised KCOT specimens (Figure 1A). The genotypes were correlated to the phenotypes, with reference to clinical, histological and immunohistological parameters. Our results indicated a significant role of *SUFU* as well as *PTCH1*, in contrast to a minor role of *PTCH2*, in the pathogenesis of KCOT. Furthermore, comparison across the genotype-oriented subgroups revealed that each has distinct clinicopathological features.

## Materials and Methods

### Patients

Thirty-six Japanese patients with KCOT who visited the Tokyo Medical and Dental University Hospital were enrolled in this study. They were 20 males and 16 females whose ages at first visit to our clinic ranged from 10 to 81 years, with a median of 32.0 years. Sixteen patients fulfilled the diagnostic criteria of GS [12], and they are referred to as hereditary KCOT patients hereafter. The remaining 20 patients, who had none of the symptoms of GS other than KCOT, are referred to as sporadic KCOT patients hereafter. All of the sporadic KCOT patients had a solitary lesion. The hereditary KCOT patients had 2.8 lesions on average (range, 1–7 lesions). The age of hereditary KCOT patients (median, 17.0 years) was significantly lower than that of sporadic KCOT patients (median, 57.5 years).

All experiments were performed according to the protocols that were reviewed and approved by the ethical committee of Tokyo Medical and Dental University. For those agreeing to participate in this study, written consent was obtained from patients 20 years of age or older, and from the parent of minor children.

### DNA Extraction

Buccal swab or peripheral blood samples were collected from the patients and from 10 healthy volunteers. DNA extraction from buccal swab samples was carried out using a QIAamp DNA FFPE Tissue Kit (Qiagen, CA, USA). DNA extraction from blood was performed using a Wizard Genomic DNA Purification Kit (Promega, WI, USA). Formalin-fixed, paraffin-embedded surgical specimens were cut into 12 µm thick sections and deparaffinized, and the lining epithelium (tumor cells) and the fibrous connective tissue (normal cells) were separately microdissected using a 29-gauge needle. Tissues with inflammatory infiltrate were excluded to avoid contamination of lymphocytes. DNA was extracted using a QIAamp DNA FFPE Tissue Kit (Qiagen).

### PCR and DNA Sequencing

Genomic fragments containing coding exons and flanking introns of *PTCH1* (exon 1∼23), *PTCH2* (exon 1∼22) and *SUFU* (exon 1∼12) were obtained by PCR using primers listed in Table S1. PCR was performed using a total volume of 30 µL containing about 1 ng of template DNA, 6.25 µmol dNTPs, 20 pmol of each primer, 0.25 U of PrimeSTAR GXL DNA polymerase (TaKaRa Bio, Shiga, Japan). All PCR amplifications were done at 95°C for 2 min, followed by 32 cycles of 94°C for 30 sec, 60°C for 30 sec and 72°C for 30 sec. PCR products were purified using a MonoFas DNA purification Kit (GL Sciences, Tokyo, Japan). DNA sequencing was performed using BigDye Terminator v1.1 Cycle Sequencing Kit and ABI PRISM 3130×l Genetic Analyzer (Life Technologies, CA, USA). Identified mutations were compared to the registered sequences on the Human Gene Mutation Database (http://www.hgmd.org/).

### LOH Analysis

Primer sequences of the microsatellite markers are listed in Table S1. One of each primer pair was 5′-labeled with FAM. PCR was performed separately on DNA samples obtained from tumor cells and normal cells. After confirming correct amplification by agarose gel electrophoresis, the PCR products were analyzed using ABI PRISM 3130×l Genetic Analyzer (Life Technologies). The ratios of short allele-normal (Sn) versus short allele-tumor (St) and long allele-normal (Ln) versus long allele-tumor (Lt) were calculated, and more than 50% difference between St/Sn and Lt/Ln was regarded as LOH. When all the microsatellite markers were homozygous in the normal cells, the case was considered non-informative. LOH of at least one microsatellite marker was regarded as LOH at the corresponding allele.

### Histological and Immunohistological Analyses

All the specimens were reevaluated and the diagnoses of KCOT were confirmed. Epithelial budding, epithelial islands and daughter cysts were checked according to the criteria described in the literature [32]. Epithelial budding is distinct mural invagination from the basal layer into the underlying fibrous connective tissue. An epithelial island is a detached lump of epithelial cells. A daughter cyst is a small cyst full of keratin squames, which locates far from the main cystic cavity. Two examiners independently assessed the microscopic findings and confirmed each evaluation. Immunohistochemical staining was performed as previously described [33] using anti-GLI1 (H-300, Santa Cruz Biotechnology, CA, USA), GLI2 (H-300, Santa Cruz Biotechnology), PTCH1 (C53A3, Cell Signaling Technology, MA, USA), PTCH2 (N-19, Santa Cruz Biotechnology), SUFU (C81H7 and C54G2, Cell Signaling Technology), Cyclin-D1 (SP4, Nichirei Biosciences, Tokyo, Japan), BCL2 (124, Dako, Glostrup, Denmark) and FOXM1 (D12D5, Cell Signaling Technology) antibodies.

### Statistical Analysis

Statistical analysis was performed using the chi-square test or Fisher’s exact test where appropriate. Probability values less than 0.05 were considered statistically significant.

## Results

### Mutational Analysis of *PTCH1*, *PTCH2* and *SUFU* in KCOT Patients

We sequenced all the coding exons of *PTCH1*, *PTCH2* and *SUFU* and found 12 heterogeneous mutations of *PTCH1* in 9 patients. All 9 were hereditary KCOT patients. Cases 25, 26 and 27, which were in the same family, had two mutations. Cases 35 and 36 were not relatives as far as we could determine, but they had the same mutation (Table 1). Thus, 9 of 16 (56%) hereditary KCOT patients or 7 of 12 (58%) hereditary KCOT pedigrees carried the *PTCH1* mutations. There were 5 missense mutations in 5 pedigrees, 2 frameshift/deletion mutations in 2 patients, and a nonsense mutation in one patient (Table 1). To the best of our knowledge, 4 *PTCH1* mutations (2223C>T, 3953C>T, 1590_1600del, 2180delT) were novel ones (Figure 1B). One patient (Case 36) had a novel *PTCH2* missense mutation (524G>T), but the significance of this mutation was indefinite because the patient also had a *PTCH1* missense mutation that was identical to another unrelated patient (Case 35). The pedigrees of GS families and the representative electropherograms are shown in Figures 1C and 1D. The mutations were more frequent in the intracellular region (Figure 1B). One was in the putative sterol-sensing domain, but a mutational hot spot was not evident. We also checked genotype-phenotype correlations and we observed that, in comparison with Case 33, Case 31 had a larger deletion including the sterol-sensing domain, and it had one more major symptom–calcification of falx cerebri, implying that amino acid 622–745 may associate with calcification of falx cerebri in the deletion mutant. Correlations between the gene mutation and the clinical phenotype were not evident, and no mutation was found in the *SUFU* gene in any patients.

**Table 1:** Summary of genetic alterations and clinicopathological features of KCOT patients.

Case	Age	Sex	Mutation (Coding exon)				LOH			Phenotype												
										KCOT								BCC	Skin pit	Rib anomaly	Calcification of falx cerebri	Family with GS
			SUFU		PTCH1	PTCH2	10q24 (SUFU)	9q22-q31 (PTCH1)	1p34 (PTCH2)	GLI2	CCND1	BCL2		Epithelial budding	Epithelial island	Daughter cyst	Recurrence					
1	16	M	NI		NI	NI	−	+	−	N	S	BS		−	−	+ (1)	−	−	−	−	−	−
2	23	F	−		−	−	−	−	−	N	BS	B		−	−	−	−	−	−	−	−	−
3	23	M	−		−	−	−	−	−	N	BS	BS		−	−	−	F	−	−	−	−	−
4	25	M	−		NI	−	−	+	−	N	BS	NI		−	−	−	−	−	−	−	−	−
5	29	M	−		NI	NI	−	−	−	C	S	B		−	−	−	−	−	−	−	−	−
6	29	F	−		−	−	−	−	−	N	S	BS		−	−	−	−	−	−	−	−	−
7	32	M	−		−	−	−	−	−	C	S	B		−	−	−	−	−	−	−	−	−
8	47	M	−		−	−	−	−	−	C	S	B		−	−	−	−	−	−	−	−	−
9	50	F	−		−	−	NI	NI	NI	C	NI	NI		−	−	−	−	−	−	−	−	−
10	57	F	−		−	−	−	−	+	N	S	B		−	−	−	−	−	−	−	−	−
11	58	F	−		−	−	−	−	−	N	S	BS		+	−	+ (1)	−	−	−	−	−	−
12	59	F	−		−	NI	−	−	+	N	BS	BS		−	−	−	−	−	−	−	−	−
13	61	M	−		−	−	−	+	−	N	BS	BS		+	−	−	+	−	−	−	−	−
14	65	M	−		−	−	−	−	−	N	S	BS		−	−	−	+	−	−	−	−	−
15	66	F	−		−	NI	+	−	+	N	BS	BS		+	+	−	F	−	−	−	−	−
16	68	M	−		−	NI	−	−	+	N	NI	NI		−	−	−	−	−	−	−	−	−
17	69	M	NI		NI	NI	−	−	−	C	S	B		−	−	−	−	−	−	−	−	−
18	75	M	−		−	−	+	−	−	N	BS	BS		+	−	+ (1)	−	−	−	−	−	−
19	79	M	NI		NI	NI	−	−	−	N	S	BS		−	−	−	−	−	−	−	−	−
20	81	M	−		−	−	−	−	−	C	S	BS		−	−	−	−	−	−	−	−	−
21 *^1^	20	M	−		−	−	+	+	+	N	BS	BS		+	+	+ (6)	−	+	+	−	−	+
22 *^1^	45	F	−		−	−	NI	NI	NI	N	NI	NI		−	+	−	−	−	+	+	+	+
23 *^2^	10	F	−		−	−	**−**	**−**	**−**	N	BS	BS		+	+	+ (3)	−	−	+	+	+	+
24 *^2^	13	F	−		−	−	+	+	+	N	BS	NI		+	−	+ (2)	−	−	+	−	+	+
25 *^3^	10	F	−	2223C>T (A741V)		−	−	+	−	N	S	BS	+		−	−	+	−	+	+	−	+
				3953C>T (P1318L)																		
26 *^3^	13	F	−	2223C>T (A741V)		−	+	+	+	N	BS	BS	+		−	−	−	−	+	+	−	+
				3953C>T (P1318L)																		
27 *^3^	35	F	−	2223C>T (A741V)		−	+	+	−	N	S	BS	+		−	−	+	+	+	+	+	+
				3953C>T (P1318L)																		
28	10	M	−	387G>A (W129X) **		−	−	+	−	N	BS	BS	+		−	−	+	−	+	−	−	−
29	10	F	−	−		−	+	+	−	N	BS	BS	−		−	−	−	−	−	+	−	−
30	14	M	−	3583A>T (T1195S) **		−	−	−	−	N	BS	B	−		+	−	F	+	−	−	−	−
31	15	M	−	1590_1600del11 (I531fsX622)		−	−	+	−	N	S	BS	+		+	−	−	−	+	+	+	−
32	19	M	−	−		−	NI	NI	NI	N	S	BS	+		+	−	−	+	+	−	−	+
33	19	F	−	2180delT (C727fsX745)		−	−	+	−	N	S	BS	−		+	−	−	−	+	+	−	−
34	34	F	−	NI		−	−	−	−	N	BS	BS	+		+	−	+	+	+	−	+	+
35	38	M	−	3395T>C (S1132P) **		−	+	+	−	N	S	BS	+		−	+ (3)	+	−	+	+	−	+
36	65	M	−	3395T>C (S1132P) **		524G>T (R175L)	−	+	−	N	BS	BS	+		+	+ (7)	−	+	+	−	−	+
Total			0	9		1	8	14	7					16	10	8	7	6	14	9	6	11

Table footnotes are as follows: *^(Number)^, Pedigree number; **, Previously-reported mutation; +, Positive; -, Negative; NI, Non-informative; N, Nuclear; C, Cytoplasmic; B, Basal; S, Suprabasal; BS, Basal and suprabasal; (Number), Number of daughter cyst; F, Treated by fenestration, not enucleation.

In summary, our patients harbored only *PTCH1* mutations, except for one who also had a *PTCH2* mutation, and there were no cases indicating that a germline mutation of *PTCH2* or *SUFU* has a role in KCOT development. Sporadic KCOT patients had none of the gene mutations, which suggest that they are not genetically predisposed to KCOT or any other tumors.

### LOHs at the *PTCH1*, *PTCH2*, and *SUFU* Loci in KCOT

To examine the contribution of the allelic loss of *PTCH1*, *PTCH2*, and *SUFU* to KCOT development, tumor cells and normal cells were separately collected from surgical specimens, and LOH analysis was conducted using the short tandem repeat polymorphic markers flanking each gene locus. PCR was successfully done from DNA extracted from formalin-fixed paraffin-embedded tissue in 33 out of 36 specimens. The representative electrophoregrams displaying LOH are shown in Figure 1E. LOH of at least one gene locus was observed in 11 cases of hereditary KCOT (79%) and 8 of sporadic KCOT (42%) (Table 1). The most frequently affected locus was 9q22-q31 (*PTCH1*) (42%), followed by 10q24 (*SUFU*) (24%) and 1p34 (*PTCH2*) (21%). LOHs at *PTCH1* and *SUFU* were more frequently observed in hereditary KCOT than in sporadic KCOT (P = 0.0005 and P = 0.04, respectively). The occurrence of LOH at *PTCH2* showed no significant difference between hereditary and sporadic KCOT (P = 0.68).

In summary, LOH was most frequent in the *PTCH1* locus, followed by *SUFU* and then *PTCH2*. Compared to sporadic KCOT, hereditary KCOT exhibited more LOH at the *PTCH1* and *SUFU* loci.

### Constant Nuclear Localization of GLI2 in KCOT with Genetic Alterations

**Figure 2:**
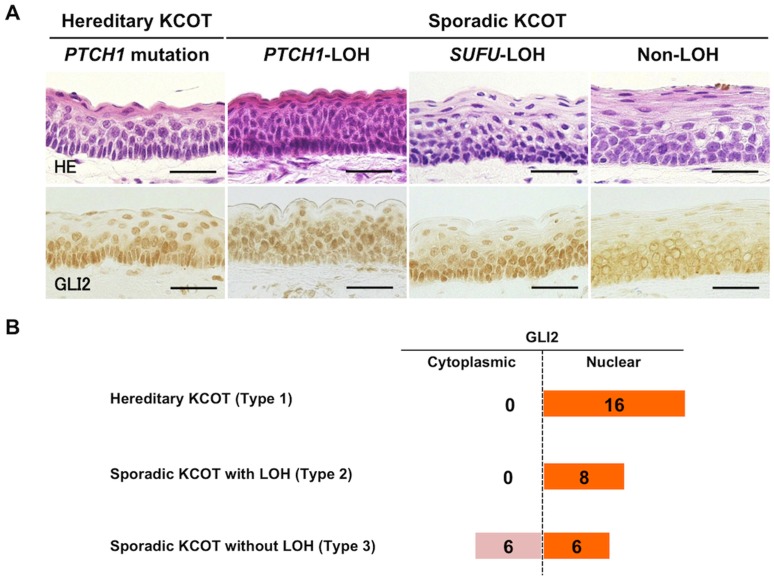
Immunohistochemical expression of GLI2 in KCOT. A) In hereditary KCOT and sporadic KCOT with LOH, GLI2 was detected in the nuclei, whereas it was detected mainly in the cytoplasm in about a half of sporadic KCOT without LOH. Scale bars = 30 µm B) Schematic table of the GLI2 expression patterns in each subgroup. Number of cases with the designated expression patterns are depicted (see also Table 1).

**Figure 3:**
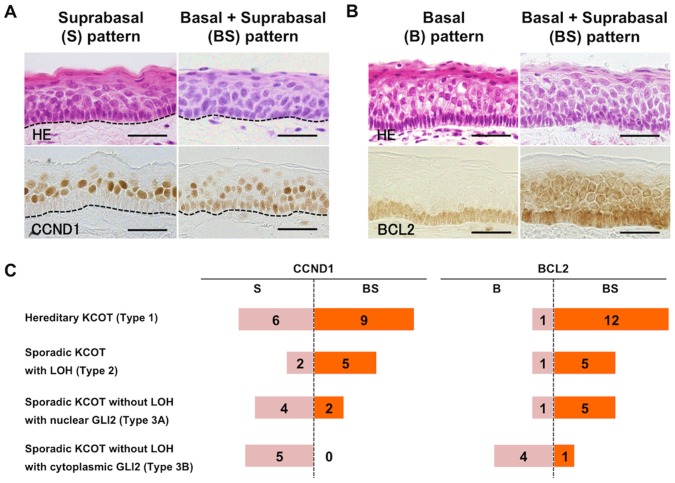
Immunohistochemical expression of CCND1 and BCL2 in KCOT. A) CCND1. Each case was classified either as the basal and suprabasal (BS) pattern or the suprabasal-dominant (S) pattern. The basal-dominant (B) pattern was not observed. The dotted line indicates a basement membrane. Scale bars = 30 µm. B) BCL2. Each case was classified either as the basal and suprabasal (BS) pattern or the basal-dominant (B) pattern. The suprabasal-dominant (S) pattern was not observed. Scale bars = 30 µm. C) Schematic table of the CCND1 and BCL2 expression patterns in each subgroup. Number of cases with the designated expression patterns are depicted (see also Table 1). The cases in which the examiners’ evaluation split were excluded.

To explore the correlation between the genetic alteration and the protein expression, we examined immunohistological expression of PTCH1, PTCH2 and SUFU in KCOT. To assess the activity of hedgehog signaling pathway, we also examined GLI1, GLI2, and their putative downstream targets, CCND1, FOXM1 and BCL2. GLI2 was expressed in all KCOT samples, and there was no apparent correlation between the level of expression and the presence of gene mutation or LOH. However, we found a correlation between the localization of GLI2 protein and the presence of LOH. The intracellular localization of GLI2 was evaluated in each case and scored either as nuclear (N) or cytoplasmic (C)(Figure 2A, Table 1). In all 8 sporadic KCOT with at least one LOH, GLI2 was observed predominantly in the nucleus (8/8, 100%, Figure 2B, Table 1). In contrast, in 6 out of 12 sporadic KCOT without LOH, GLI2 was observed mainly in the cytoplasm (6/12, 50%). The remaining 6 sporadic KCOT without LOH showed nuclear localization of GLI2. All hereditary KCOT showed nuclear localization of GLI2 (Figure 2B, Table 1). CCND1 and BCL2 were expressed in all KCOT. Since the expression patterns were different across cases, we evaluated the expression patterns of CCND1 and BCL2 in each case. The presence of positive cells was examined separately in the basal (B) and the suprabasal (S) layers, and the dominant pattern was scored either as B (basal-dominant), S (suprabasal-dominant) or BS (basal and suprabasal) (Figure 3A, 3B, Table 1). As for CCND1, sporadic KCOT without LOH tended to show the S pattern, while the other types of KCOT mostly showed the BS pattern, indicating that CCND1 expression in the basal layer was weak in sporadic KCOT without LOH (Figure 3C). Furthermore, all the cases with cytoplasmic GLI2 showed the S pattern of CCND1 (Figure 3C). BCL2 was expressed ubiquitously in the basal layer (Figure 3B). Most cases with cytoplasmic GLI2 showed the B pattern of BCL2, while the other types of KCOT were often accompanied with the BS pattern (Figure 3C). FOXM1 was expressed in the parabasal layer and less frequently in the basal layer (Figure S1). There was no significant difference across the cases in the expression patterns of FOXM1. Immunohistochemical expression of PTCH1, PTCH2 and SUFU was ubiquitously observed in KCOT, and the staining intensities and localizations did not significantly differ and did not correlate with the presence of a mutation or LOH (Figure S2). Immunohistological examination using the GLI1 antibody yielded either no staining or insignificant staining even in control tissues, which we concluded was a weak non-specific reaction (our unpublished observation).

### LOH at *PTCH1* and *SUFU* Loci Pertains to Epithelial Budding, which Predisposes for Recurrence

LOHs at chromosome 1p and 9q are correlated with histological subtypes in glioma and medulloblastoma, respectively, and they can mold a histological and clinical phenotype [34], [35]. We speculated that the LOHs were correlated to the histological and clinical features of KCOT, and we checked this hypothesis. Although all KCOT shares common histological features, variations in histological patterns, such as daughter cysts, epithelial islands and epithelial budding, are occasionally observed (Figure S3). We assessed these variables and examined the correlation with LOH. LOHs at *PTCH1* and *SUFU* loci were significantly associated with the presence of epithelial budding (Table 2), while *PTCH2* LOH was not. No correlation was observed between the presence of epithelial islands or daughter cysts and LOH at any loci (all P>0.05).

**Table 2:** Correlation between the presence of each LOH and the histological parameters.

LOH		Epithelial budding		P value	Epithelial island		P value	Daughter cyst		P value
		present	absent		present	absent		present	absent	
9q22-q31 (*PTCH1*)	present	10	4	0.013	4	10	0.46	5	9	0.18
	absent	5	14		4	15		3	16	
1p34 (*PTCH2*)	present	4	3	0.39	2	5	0.47	2	5	0.56
	absent	11	15		5	21		6	20	
10q24 (*SUFU*)	present	7	1	0.0088	2	6	0.56	4	4	0.074
	absent	8	17		5	20		4	21	

LOHs at the *PTCH1* and *SUFU* loci were significantly associated with the presence of epithelial budding, while *PTCH2* LOH was not.

No correlation was observed between the other histological features and LOH at any loci.

Average ages of patients with and without *PTCH1* LOH were 25 and 48 years, respectively, suggesting that *PTCH1* LOH was significantly correlated with early onset of the lesion (P = 0.005), while *PTCH2* (P = 1.00) and *SUFU* (P = 0.72) were not. No correlation was observed between the presence of any LOH and the size of tumor. Post surgery recurrence was observed in seven of the total 33 KCOT that were treated by enucleation and followed for more than a year. Although the recurrence was not correlated with any LOH, it was significantly correlated with the presence of epithelial budding (P = 0.014), but not with the presence of daughter cysts (P = 0.53) or epithelial islands (P = 0.36). Finally, epithelial budding (P = 0.001) and epithelial islands (P = 0.001) were more frequently observed in hereditary lesions.

In summary, LOHs at *PTCH1* and *SUFU* loci pertain to epithelial budding, which predisposes for recurrence.

## Discussion

We engaged in mutational analysis of sporadic KCOT patients to check the hypothesis that they may be predisposed to KCOT because of germline mutations of *PTCH1*, *PTCH2* or *SUFU*, whose phenotypes are too weak to present with any other symptoms. Against our expectations, none of the sporadic KCOT patients harbored a gene mutation. This indicates that normal follow-up for local recurrence is sufficient as clinical management of sporadic KCOT patients.

We saw a high incidence of *PTCH1* mutations in our hereditary KCOT patients, all of whom met the diagnostic criteria for GS, and we could demonstrate four novel mutations, while no mutation was found in the *PTCH2* or *SUFU* gene. These results confirm the predominance of *PTCH1*-associated GS families in the current human population. A germline mutation of *SUFU* has been reported only in 6 patients, 5 of whom developed medulloblastoma [30]. Although the incidence of medulloblastoma appears higher in *Ptch1* ^+/−^ mice than Sufu^+/−^ mice [36], [37], haploinsufficiency of *SUFU* in humans seemingly leads to a stronger predisposition to medulloblastoma [38] compared to haploinsufficiency of *PTCH1*, in which the patients develop medulloblastoma at less than 10% frequency [39]. This malignant brain tumor mainly arises in children. Unlike people affected by BCC or KCOT, most untreated medulloblastoma patients die before reaching reproductive age. Natural selection due to the high incidence of lethal medulloblastoma might have decreased the *SUFU*-mutant pedigrees more than the *Ptch1*-mutant pedigrees.

KCOT exhibited a variety of genetic alterations, and we propose to divide them into three groups: 1) KCOT with a germline mutation in the hedgehog-related gene; 2) KCOT with LOH but without a germline mutation; and 3) KCOT with neither a germline mutation nor LOH. We have designated these as Types 1, 2 and 3. Our data clearly demonstrated that all Type 1 KCOT developed with systemic abnormalities and were diagnosed with GS. Gene mutations were not detected in six GS patients. Among these, five were familial cases, indicating that they must have a pathogenic gene alteration in the germline, and implying that these can be regarded as putative Type 1 cases, although the mutation was not specified in the scope of the present investigation. Only one case lacked evidence of a germline gene alteration since it was a non-familial case, and our effort to find a gene mutation failed.

Besides the classic two-hit mechanism, a tumor can arise when only one allele of the tumor suppressor gene is affected [18], [40]. For example, medulloblastoma and BCC-like skin tumors in *Ptch1* ^+/−^ or *Sufu* ^+/−^ mice can retain the wild-type allele of the genes [31], [36], [41], [42]. This gene-dosage effect, namely haploinsufficiency, is thought to be an important mechanism for tumor development [40]. In fact, the cases that met the two-hit model, in which *PTCH1* LOH and mutation coexisted, constituted only half of the Type 1 lesions.

In Type 1 and Type 2 lesions, genetic insult in the hedgehog signaling pathway is highly suspected as the etiology. This notion is supported by the consistent observation of GLI2 nuclear localization in these lesions. Gli is a dual-functional transcription factor. In the absence of hedgehog signal input, Gli is proteolytically processed into a transcriptional repressor. When the hedgehog signaling pathway is activated, Gli is converted into a transcriptional activator and it translocates to the nucleus [6]. *Gli2* transgenic mice have developed BCC-like skin tumors and keratinizing jaw cysts, indicating that Gli2 overexpression is sufficient for jaw cyst formation [43], [44]. Gli1 is another well-defined hedgehog signaling effector, but there is no report on occurrence of jaw cysts in *Gli1* transgenic mice, suggesting that GLI2 is more essential than GLI1 in KCOT development. In addition, we found substantial expression of several GLI2 downstream targets, such as CCND1, BCL2 and FOXM1, in KCOT. Collectively, Type 1 and Type 2 are characterized by evident indication of both the genetic alteration and the activation of hedgehog signaling pathway.

In contrast, Type 3 is a lesion in which evidence of genetic insult on the hedgehog pathway is lacking. Type 3 may consist of two different entities, namely Type 3A and Type 3B. Type 3A exhibited the nuclear accumulation of GLI2, suggesting that the hedgehog pathway was activated like Type 1 and Type 2, probably by a tumor cell-specific somatic mutation. This can be checked by DNA sequencing of KCOT cells. However, the quality of DNA obtained from formalin-fixed paraffin embedded specimens was not sufficient to obtain all the necessary PCR products for comprehensive sequencing (data not shown). Microdissection of KCOT cells from a fresh tissue sample is necessary to investigate the somatic mutation. Type 3B presented the cytoplasmic localization of GLI2, the reduced expression of CCND1 in the basal layer and the reduced expression of BCL2 in the suprabasal layer, suggesting a low activity of the hedgehog signaling pathway (Figure 3). It should be noted that FOXM1 was expressed in Type 3B in a similar fashion to the other types. This may suggest that the low activity of the hedgehog signaling pathway is enough for the upregulation of FOXM1.

Formation of epithelial budding, epithelial islands and daughter cysts appears to involve different cell behaviors from those required for cyst formation. Cyst would be formed solely by proliferation of the basal cells with accumulation of cyst contents pushing the wall outward. In contrast, formation of epithelial islands and daughter cysts would need invasive protrusion of KCOT nests into the surrounding connective tissue. This tendency of protrusion into the stromal tissue is reminiscent of early stages of tooth germ formation that is initiated with invagination of primitive oral epithelium. Epithelial budding in KCOT may also recapitulate this initial stage of anlage development. We assume that these histological features may represent the invasive potential of KCOT. Interestingly, the incidences of these histological features tended to correlate with the types of KCOT. Epithelial budding was observed at a significantly higher frequency in Type 1 compared with Type 2 and Type 3, and it was more frequent in Type 2 than in Type 3. Also, epithelial islands and daughter cysts were more frequent in Type 1. Notably, we did not find any of these histological features in Type 3B, which is in line with the fact that no recurrence was detected in the Type 3B cases.

The presence of LOH at the *PTCH1* and *SUFU* loci was correlated with a tendency toward epithelial budding, whereas *PTCH2* was not. *Sufu* ^+/−^ [31], [45], [46] and *Ptch1* ^+/−^ [45], [47] mice had a unique histological phenotype of the skin, showing basal cell hyperplasia and elongated rete-ridge-like protrusions that are reminiscent of epithelial budding. This skin phenotype was observed in the *Ptch1* ^+/−^ mice with 30% penetrance [45], [47], and in the *Sufu* ^+/−^ mice with 100% penetrance [31], [46], suggesting that haploinsufficiency of these genes leads to aberrant regulation of rete ridge morphogenesis. On the contrary, *PTCH2* knockout mice did not have any significant phenotypes [48]. We speculate that the contribution of *PTCH2* to KCOT development may not be large compared to *PTCH1* and *SUFU*.

**Figure 4:**
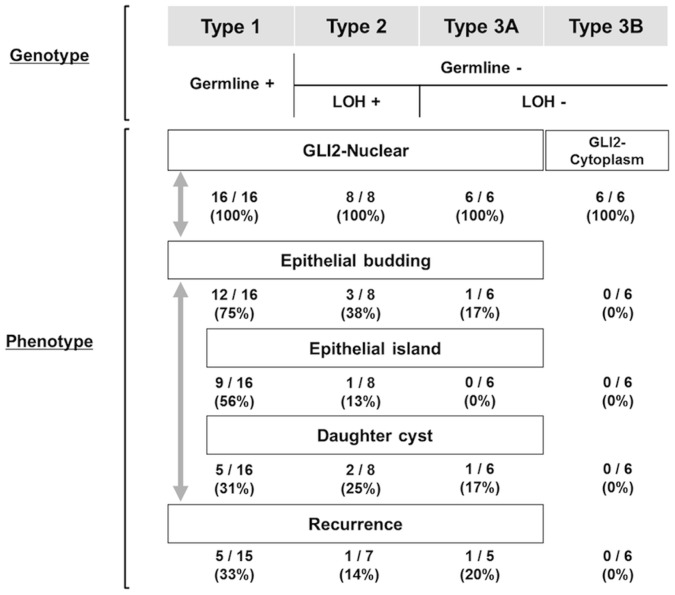
Schematic summary of this study. KCOT can be divided into three groups by genotype. Type 1 is KCOT with a germline mutation. Type 2 is KCOT with LOH but without a germline mutation. Type 3 is KCOT with neither a germline mutation nor LOH. Type 3 consists of two subtypes: Type 3A, with nuclear localization of GLI2; and Type 3B, with cytoplasmic localization of GLI2. Epithelial budding and recurrence are noted most frequently in Type 1 and least frequently in Type 3B.

Figure 4 summarizes the geno-phenotype correlation in KCOT. KCOT with a germline mutation (Type 1) showed nuclear GLI2 accumulation and frequent findings of epithelial budding, epithelial islands and daughter cysts, and the highest recurrence rate. KCOT with LOH but without a germline mutation (Type 2) also presented nuclear GLI2 accumulation, a less frequent occurrence of the abovementioned findings, and a lower recurrent rate compared to Type 1. KCOT with neither a germline mutation nor LOH (Type 3) seemingly consists of two entities. Type 3B lesions exhibited cytoplasmic localization of GLI2, and epithelial budding, epithelial islands, daughter cysts and recurrence were rare, constituting the most amicable entity. The term ‘tumor’ might not be appropriate for Type 3B lesions.

In conclusion, our data demonstrated that KCOT consists of genetically heterogenous entities. Alteration of *SUFU* as well as *PTCH1* is associated with distinct clinicopathological features. The subgroups on the basis of the presence of gene mutation and LOH constitute entities with different potential aggressiveness, suggesting that the biological behavior of KCOT may be predicted more accurately by dividing it into genotype-oriented subtypes.

## Supporting Information

Figure S1 **Immunohistochemical expression of FOXM1 in KCOT.** There was no significant difference in the FOXM1 expression pattern across the cases. Scale bars = 30 µm.
(TIF)

Figure S2 **Immunohistochemical expression of PTCH1, PTCH2 and SUFU in KCOT.** The staining intensities and localizations did not significantly differ and did not correlate with the presence of mutations or LOH. Scale bars = 30 µm.
(TIF)

Figure S3 **Histology of KCOT.** A) A neoplastic parakeratinized squamous epithelium has a flat interface with the connective tissue. Scale bar = 30 µm. B) Epithelial budding. The neoplastic epithelium extends toward the fibrous connective tissue. Scale bar = 30 µm. C) Epithelial island. Detached lumps of epithelial cells in the fibrous connective tissue (arrows). Scale bar = 60 µm. D) Daughter cyst. A small cyst that is separated from the main cyst cavity. Scale bar = 200 µm.
(TIF)

Table S1 **List of PCR primers.**
(DOC)
