# Supplementary figures and images for: Integrated Genotypic Analysis of Hedgehog-Related Genes Identifies Subgroups of Keratocystic Odontogenic Tumor with Distinct Clinicopathological Features
Source: PLoS One. 2013 Aug 7;8(8):e70995. doi: 10.1371/journal.pone.0070995 (PMC3737235; doi:10.1371/journal.pone.0070995)

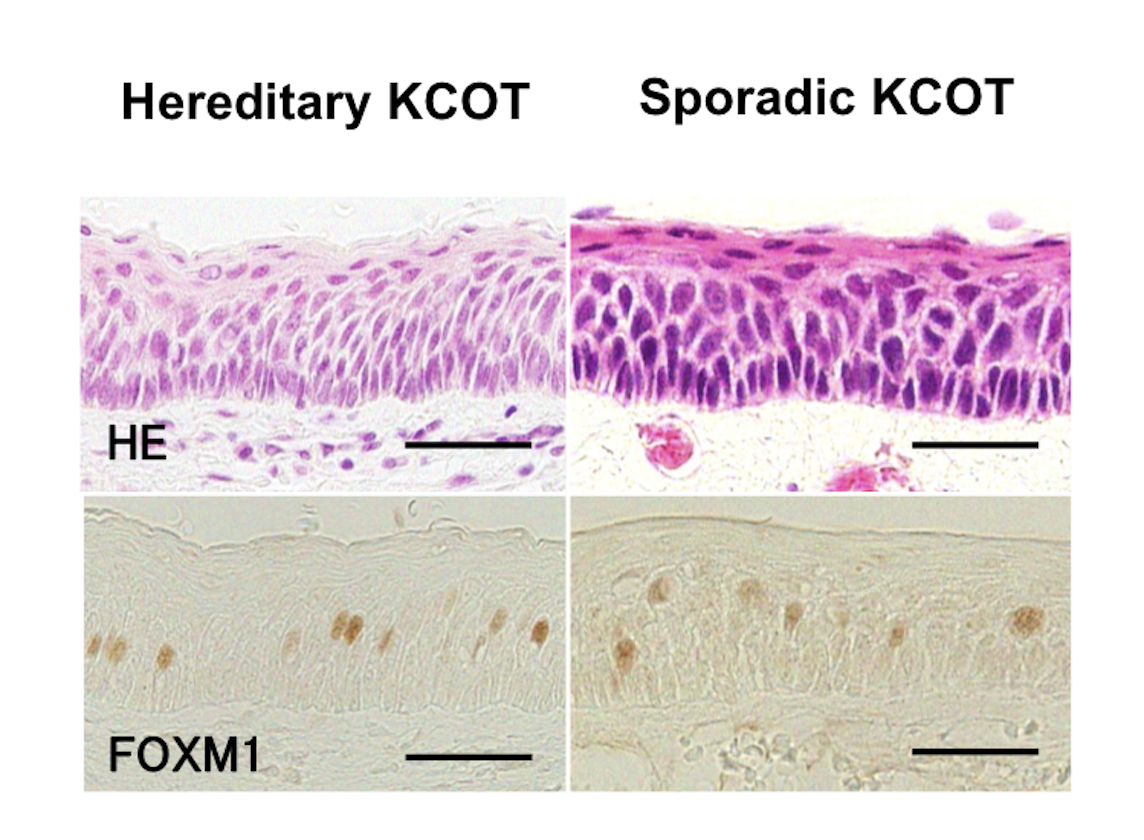

Supplement: Figure S1 — Immunohistochemical expression of FOXM1 in KCOT. There was no significant difference in the FOXM1 expression pattern across the cases. Scale bars = 30 µm. (TIF) [file pone.0070995.s001.tif]

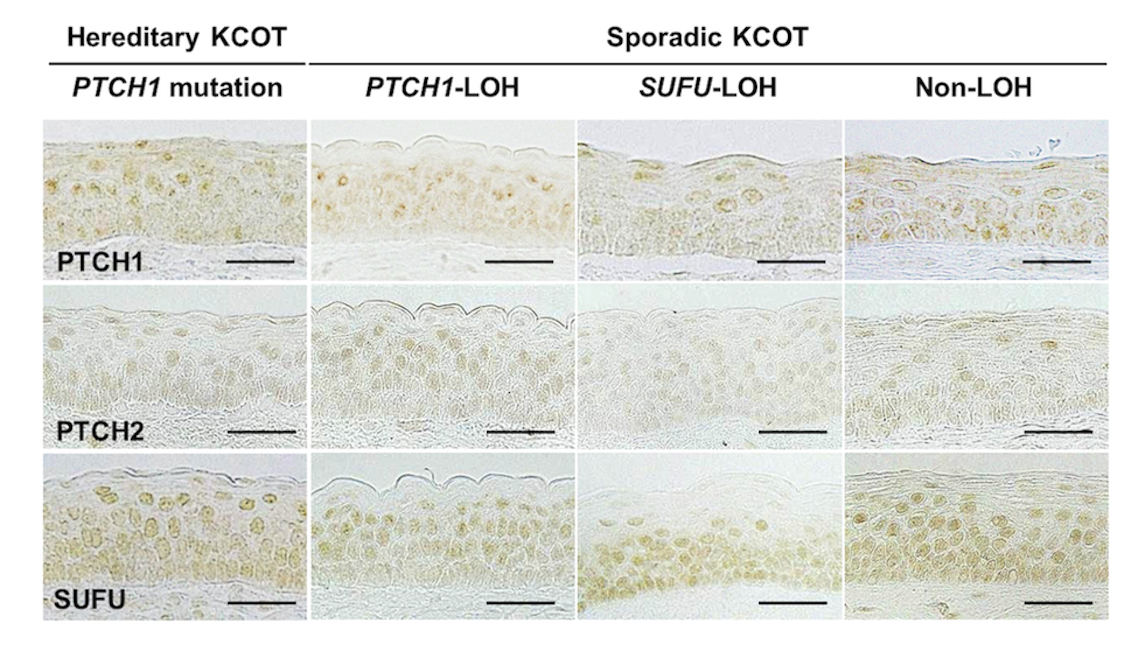

Supplement: Figure S2 — Immunohistochemical expression of PTCH1, PTCH2 and SUFU in KCOT. The staining intensities and localizations did not significantly differ and did not correlate with the presence of mutations or LOH. Scale bars = 30 µm. (TIF) [file pone.0070995.s002.tif]

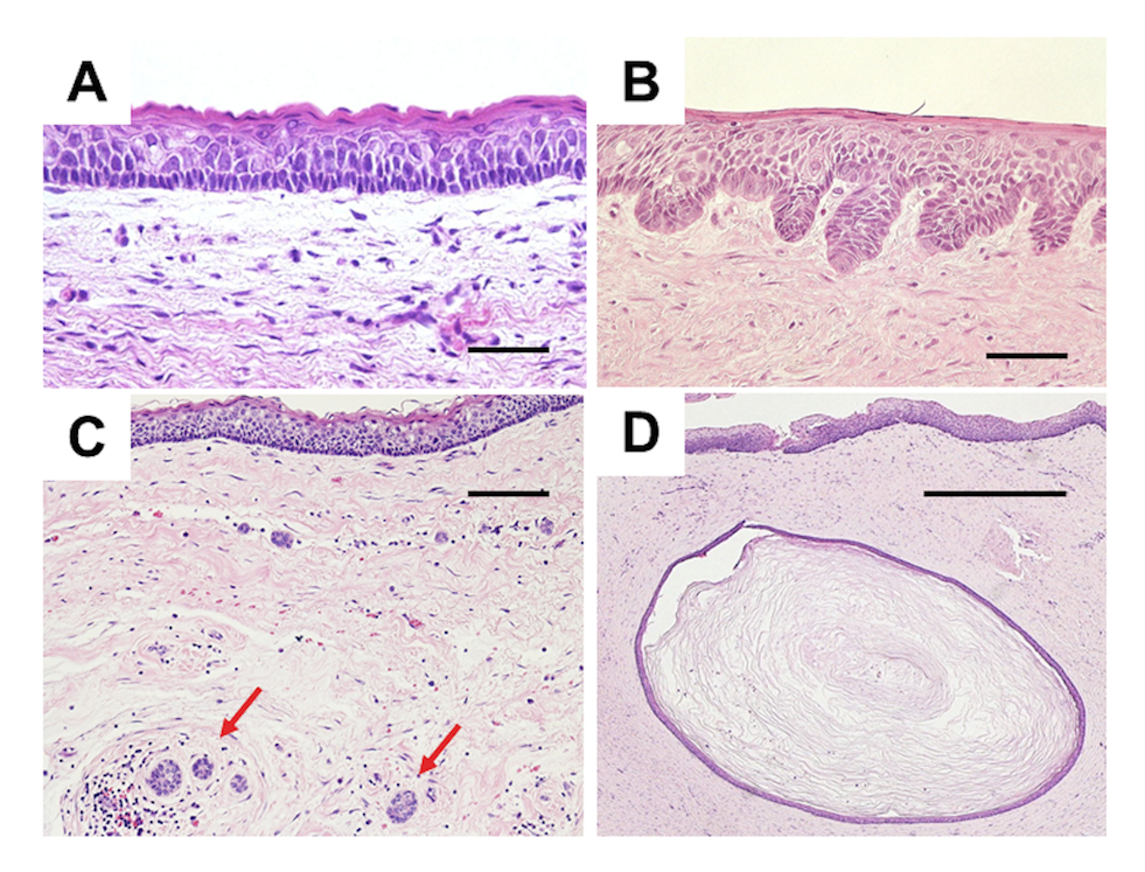

Supplement: Figure S3 — Histology of KCOT. A) A neoplastic parakeratinized squamous epithelium has a flat interface with the connective tissue. Scale bar = 30 µm. B) Epithelial budding. The neoplastic epithelium extends toward the fibrous connective tissue. Scale bar = 30 µm. C) Epithelial island. Detached lumps of epithelial cells in the fibrous connective tissue (arrows). Scale bar = 60 µm. D) Daughter cyst. A small cyst that is separated from the main cyst cavity. Scale bar = 200 µm. (TIF) [file pone.0070995.s003.tif]
