# Supplementary material for: Integrated Genotypic Analysis of Hedgehog-Related Genes Identifies Subgroups of Keratocystic Odontogenic Tumor with Distinct Clinicopathological Features
Source: PLoS One. 2013 Aug 7;8(8):e70995. doi: 10.1371/journal.pone.0070995 (PMC3737235; doi:10.1371/journal.pone.0070995)
Supplement: Table S1 — List of PCR primers. (DOC) [file pone.0070995.s004.doc]

| Table S1; List of PCR primers | | | |
| --- | --- | --- | --- |
| *PTCH1*  Exon | Forward primer (5'-3') | Reverse primer (5'-3') | Size of PCR product (bp) |
| 1 | AAGGCGCAGGGTCTGACT | GGGAAGAGAAAGTGGGAGGA | 476 |
| 2 | ACTCCTCCCTTCTGCTTCGT | ATCTCTATCAACCGCGAGGA | 282 |
| 3 | GAGTTTGCAGTGATGTTGCTATTC | ACCGCCTTACCTGCTGCTC | 291 |
| 4 | TGCACTAATTTTCTTATTACAGTGAG | TAAGGCACACTACTGGGGTG | 184 |
| 5 | GAACACCCCAGTAGTGTGCC | TGAGTCCTAGAGAAGTCACAGACATC | 200 |
| 6 | GGCTCTTTTCATGGTCTCGTC | TGTTTTGCTCTCCACCCTTC | 284 |
| 7 | GCACTGGATTTTAACAAGGCATG | AGGGCATAGATTGTCCTCCG | 240 |
| 8 | TGGGAATACTGATGATGTGCC | CATAACCAGCGAGTCTGCAC | 232 |
| 9 | CATTTGGGCATTTCGCATTC | ACCAAACCAAACTCCAGCCC | 211 |
| 10 | TGCCCCCATTGTTCTGCTTG | GGACAGCAGATAAATGGCTCC | 208 |
| 11 | GCATCTCGCATGTCTAATGCCAC | AAGCTGTGATGTCCCCAAAG | 188 |
| 12 | GACCATGTCCAGTGCAGCTC | CGTTCAGGATCACCACAGCC | 211 |
| 13 | AGTCCTCTGATTGGGCGGAG | CCATTCTGCACCCAATCAAAAG | 222 |
| 14 | AAAATGGCAGAATGAAAGCACC | CTGATGAACTCCAAAGGTTCTG | 540 |
| 15 | GGAAGAGTCAGTGGTGCTCC | CGCCAAAGACCGAAAGGAC | 405 |
| 16 | AGGGTCCTTCTGGCTGCGAG | GCTGTCAAGCAGCCTCCAC | 271 |
| 17 | GCTCTCAAGGCAGAAGTGTG | GGAAGGCACCTCTGTAAGTTC | 303 |
| 18 | GCTCCTAACCTGTGCCCTTC | GAATTTGACTTCCACAAAGCCC | 348 |
| 19 | CGCCCACTGACCACTGTGTG | GAGCCAGAGGAAATGGGTTG | 227 |
| 20 | AGCATTTACCAGGTGAAGTCC | TTGCACACGCCTGCTTAC | 217 |
| 21 | TGTTCCCGTTTCCTCTTG | GCACAGGAAACACAGCATTC | 187 |
| 22 | GCAGGTAAATGGACAAGAACAC | ACTACCACGGTGGGAAGACC | 352 |
| 23 | CCCTTCTAACCCACCCTCAC | GACACATCAGCCTTGCTC | 731 |

|  | | | | |
| --- | --- | --- | --- | --- |
| *PTCH2*  Exon | Forward primer (5'-3') | Reverse primer (5'-3') | Size of PCR product (bp) |  |
| 1 | TAACACCCACACCCCACAGT | CTCGGCACTTCAAGACATCA | 319 |  |
| 2 | TGTCCCACATCCAGAAATCA | TGGCCAGTGTCCACAACTTA | 332 |  |
| 3 | CCCAGCTTTCTCTGTTTGCT | AATAGCCAGGGGCATAGGAG | 320 |  |
| 4 | GAGTGGGAATCCCCTTCTTC | AGTCCTCCCACGCTACAGAA | 176 |  |
| 5 | TTCTGTAGCGTGGGAGGACT | GAAGCCCATGCTCTCTGTTC | 376 |  |
| 6 | TAACCCTGCCCTGATATTCG | GGAAAGGGAAGGAAAACAGC | 479 |  |
| 7 | TTGAGCATTAACCCCTCCTG | GAACAGAGTCCCCTCACCAA | 192 |  |
| 8 | CTCCTCTGGGAGTTGGTGAG | CCTTGTCCTTGTCCATACCG | 310 |  |
| 9 | CGGTATGGACAAGGACAAGG | AGGTGCCAGGTGCAAGAC | 232 |  |
| 10 | GTCTTGCACCTGGCACCT | GTGACTGGCACTGAGTCTGC | 279 |  |
| 11 | AGACTCAGTGCCAGTCACCA | TTAGCAGCCCAAGGTCACTT | 241 |  |
| 12 | TGTTATGACTGCCCCACCTC | GGAATGAAGGCTGGATGAAG | 310 |  |
| 13 | GCTTCATCCAGCCTTCATTC | GGTCTGTGCCTTGAAATGCT | 237 |  |
| 14 | AAGGCACAGACCTGTCATCC | CCTGCTCTGCCCAGTCTTAC | 407 |  |
| 15 | GGCTTAGTCCACTGCCTGAG | GGGAGACCAGGATAGGGTTC | 470 |  |
| 16 | GGGGCCACCAGCTAATAGA | AGAGGCAGAGAGGGCTGAAG | 264 |  |
| 17 | GGAGGGGTCCACTAGTACAGG | AGGCTCAGGGCTTGTGTG | 310 |  |
| 18 | ACACAAGCCCTGAGCCTGA | GTGTCTCTGTCCCCACTCCT | 404 |  |
| 19 | CAGGAGTGGGGACAGAGACA | GGAATGAGCTACCACGTCCA | 379 |  |
| 20 | TTAGTTCACCCAGCCTTTGG | CTGGGAGTAGAGGGATGGTG | 478 |  |
| 21 | ATCTCCACATTGCTGGGTCT | CAAACCCTGCACCTTGTTCT | 471 |  |
| 22 | CAGAACAAGGTGCAGGGTTT | CCACACATGGTCTCTGTGCT | 492 |  |

|  | | | |
| --- | --- | --- | --- |
| *SUFU* Exon | Forward primer (5'-3') | Reverse primer (5'-3') | Size of PCR product (bp) |
| 1 | AGCTAGACCTCGCTGCAGC | TCCCCATTCCCAACTTGAGC | 481 |
| 2 | CATCCCTTAGTCTCATTCTGG | TCCTGCACTAGATAAGCAGG | 488 |
| 3 | GGATGAGGCAAGAGTTCTGG | CAAAACAAGCCATCCTCATGG | 478 |
| 4 | CTTCCATCCGGAGTGAATGC | GACCATCTGAATCTACCTGAC | 453 |
| 5, 6 | TGGGTAGCTGACCTTCTTGG | TTTCCCAGGATGAAAGCCAG | 579 |
| 7 | CATGCTCAGCACCACAAGG | ACACAACGGTGCCTGCTGG | 512 |
| 8 | CACTCAGCGCTTACATCTGG | CCACAGATAGATCTGTGTGC | 434 |
| 9 | AGCCAGTCAGTGAGAAGTGC | AGCCTGCTTAGGTGTGTAGG | 527 |
| 10 | AGCGTGTTTGGATACAGTCC | AGGAAGTGCTCCTAAGCTGC | 509 |
| 11 | ACAGGCCTCAAACACTTCC | AGCCTATCCCTGCACCTTGG | 392 |
| 12 | GCATTTGCCCACTCACTGG | GCATTTGAGAATGAAGCCACG | 479 |

|  | | |  |  |
| --- | --- | --- | --- | --- |
| Gene locus | Name of microsatellite marker | Forward primer (5'-3') | Reverse primer (5'-3') | Size of PCR product (bp) |
| 1p34 | D1S2797 | ATCACATCACACACAATGACTGTGG | TGTCCATTCAAAGGATTGGTCTC | 144-180 |
|  | D1S2713 | CAGCCCCCAACACATAC | CCTTAGGAGTCTACAGACGCC | 227-279 |
|  | D1S421 | AGCTGGCATCTCACCC | CTGACTGGCGTTACATTCTC | 146-152 |
| 9q22-q31 | D9S287 | AGGATGCTCCTCACGC | ACCACTACATTGTTCAAGGG | 168-180 |
|  | PTCH1_exon1a | CCTCCTCTAACTCTTTGGGATCGC | GACGGCTTTCCAGTGCTCC | 264-282 |
|  | D9S1690 | GACTGGCCCGAAATTCT | CTGGCCTCCCATAAAGTT | 225-239 |
| 10q24 | D10S1738 | GGTACTGTCCTTATTTGGG | TCATCTACATTCACCAGTTG | 183-213 |
|  | D10S1697 | GCTGCTTCGATGGAAAC | AACCTGTGTCGGCTGC | 167-201 |
|  | D10S1267 | AGGATGTCAAACATAACTGGGTAG | GGAGGCTGGAGGTAAGG | 175-213 |
